# Chronic Kidney Disease Is a Risk Factor for the Development of Hyperchloremic Metabolic Acidosis After Repeated Therapeutic Plasma Exchanges

**DOI:** 10.1002/jca.70059

**Published:** 2025-10-07

**Authors:** Sanédy SA Simon, Marit S van Sandwijk, Rik H Olde Engberink

**Affiliations:** ^1^ Department of Nephrology, Amsterdam UMC University of Amsterdam Amsterdam the Netherlands; ^2^ Amsterdam Cardiovascular Sciences, Diabetes and Hypertensive Diseases Amsterdam UMC Amsterdam the Netherlands; ^3^ Dianet Dialysis Centre Amsterdam the Netherlands

**Keywords:** acidosis, chloride, chronic kidney disease, plasma exchange, plasmapheresis, renal function

## Abstract

Hyperchloremic metabolic acidosis is an underreported but common metabolic complication following therapeutic plasma exchange (TPE) with an albumin‐saline solution, particularly when multiple TPE sessions are performed within a limited period. The risk of hyperchloremic metabolic acidosis may be the highest in patients with chronic kidney disease because of their limited acid excretion capacity. We prospectively collected data from all patients who received TPE at Amsterdam UMC between February 2023 and March 2025. We collected data on demographics, TPE‐related parameters, and blood electrolyte concentrations. We investigated the effect of TPE on plasma sodium, chloride, and bicarbonate concentrations, the occurrence of adverse events, and the modulating role of kidney function. Data from 64 patients with 320 TPE sessions were included in the analysis. The mean age was 50 years, 55% of the patients were male and the mean eGFR was 35 mL/min/1.73 m^2^. The effect of a single TPE on plasma electrolyte concentration was independent of kidney function. However, after multiple TPE sessions, a lower eGFR was associated with a greater increase in plasma chloride concentration (*p* < 0.001) and a larger decrease in plasma bicarbonate concentration (*p <* 0.001) despite oral bicarbonate supplementation and a lower baseline plasma bicarbonate concentration. Patients with a lower eGFR were more likely to experience adverse events during a TPE session (*p* = 0.004). Chronic kidney disease is a risk factor for developing hyperchloremic metabolic acidosis and adverse events during an intensive TPE cycle.

## 1 Introduction

Therapeutic plasma exchange (TPE) is often performed in patients with impaired kidney function, as it can be part of the treatment of renal autoimmune diseases and renal transplant rejection. Depending on the indication for TPE, the discarded plasma is replaced by either fresh‐frozen plasma (FFP) or albumin‐saline solutions [1].

Due to the nature of TPE, this treatment can result in various metabolic disturbances, mostly related to citrate accumulation, such as hypocalcaemia and metabolic alkalosis [2]. A less known adverse effect is hyperchloremic metabolic acidosis resulting from the administration of large volumes of saline‐containing albumin solutions during the procedure. Although the exact chloride concentration is often not documented on the medication information leaflet, the chloride concentration of albumin solutions typically ranges between 109 and 145 mmol/L [3, 4].

We have recently demonstrated that patients with impaired renal function may, in particular, be susceptible to severe and symptomatic hyperchloremic acidosis [5]. This risk might be the greatest when multiple TPE sessions are performed within a limited period [6]. It is unknown whether hyperchloremic metabolic acidosis due to TPE negatively impacts short‐ or long‐term health outcomes. For instance, metabolic acidosis has been associated with headache, nausea, vomiting, and lack of energy, and has been shown to impact renal function in the long term in chronic kidney disease and renal transplant patients [7, 8, 9, 10, 11].

Therefore, we aim to investigate the effect of a single and multiple TPE sessions on the acid–base balance in patients with varying kidney function. Furthermore, we will study the clinical implications of these metabolic changes in an exploratory analysis.

## 2 Method

### 2.1 Data Collection

We prospectively collected data from all TPE procedures performed at Dianet, Amsterdam UMC location Meibergdreef, between February 1, 2023, and March 1, 2025. Amsterdam UMC specializes in TPE for both renal and non‐renal indications and operates two locations. Our study location provides TPE for nephrology, neurology, and dermatology patients, whereas most hematology patients are treated at a different location. We extracted data from the patient electronic file regarding patient demographics, medical history, use of bicarbonate supplements, TPE‐related parameters such as exchanged volume and replacement fluid, patient‐reported symptoms, and laboratory parameters including plasma sodium ([Na^+^]), chloride ([Cl^−^]), potassium ([K^+^]), albumin ([Alb^−^]), calcium ([Ca^2+^]), ionized calcium ([iCa^2+^]), bicarbonate ([HCO_3_^−^]) concentrations, and kidney function (estimated glomerular filtration rate [eGFR] CKD‐EPI 2021). Furthermore, we calculated the albumin‐adjusted anion gap (AG) before and after each session using the following formula: corrected AG = AG + 0.25 × (40 − albumin (g/L)) as stipulated by the local laboratory protocol.

The Medical Ethical Committee of Amsterdam UMC approved the study. All participants and/or their legal representatives provided written informed consent prior to inclusion in the study.

### 2.2 Patient Selection

To assess the effect of multiple TPE sessions on the acid–base balance, we selected patients with at least 5 TPE sessions who received a 4% albumin‐saline solution as replacement fluid and had a treatment intensity of ≥ 0.6 TPE sessions per day (≥ 4.2 sessions/week). We excluded patients with missing laboratory values at the start of the cycle and/or had more than three missing values during the cycle. Furthermore, we excluded patients who received intravenous bicarbonate supplementation or dialysis during the TPE cycle.

### 2.3 Therapeutic Plasma Exchange Procedures

The attending physicians made the TPE prescription, and this was not influenced by this study. All TPE sessions were carried out using the Spectra Optia apheresis device (TerumoBCT, Lakewood Co., USA). The total duration of each session varied between 1.5 and 3 h. During the TPE procedure, acid‐citrate‐dextrose formula A (ACD‐A) was used as the anticoagulant of choice. The machine automatically controlled the administration of the anticoagulant with an ACD‐A infusion rate varying from 0.8 to 1.2 mL/min/L and a blood volume‐to‐ACD‐A ratio of 12:1. Concomitant with the ACD‐A administration, calcium was supplemented as calcium‐gluconate 10% (0.23 mmol Ca^2+^/ml) depending on the plasma ionized calcium levels. In cases of plasma ionized calcium < 1.0 mmol/L, calcium gluconate supplementation was determined in consultation with the attending physician. When plasma ionized calcium levels were between 1.0 and 1.2 mmol/L, alternating volumes of 2.5 and 5 mL calcium gluconate were administered per 250‐mL albumin bottle. For plasma ionized calcium levels > 1.2 mmol/L, 2.5 mL was added to every albumin bottle.

To monitor for citrate accumulation, which can lead to metabolic acidosis, the total‐to‐ionized calcium ratio was calculated at the end of each TPE session. A ratio > 2.5 was indicative of citrate accumulation. Additionally, other potential contributors to metabolic acidosis, such as diarrhea and lactic acidosis secondary to hypovolemia, were documented.

The chloride and sodium concentrations in the 4% albumin‐saline solution used at our center (40 g/L albumin solution; Prothya Biosolutions Netherlands B.V.) were 131 and 135 mmol/L, respectively. Blood samples were collected within 15 min before starting the treatment and within 15 min after completion of treatment. All blood samples were analyzed within 30 min of collection at the central hospital laboratory. For our analysis, we used the eGFR at the start of a TPE cycle.

### 2.4 Adverse Events

The nurses recorded all symptoms reported spontaneously by patients in the electronic health records. This process was not standardized. In the exploratory analysis, we assessed all symptoms, including fatigue, muscle weakness, nausea, vomiting, headache, symptoms related to hypocalcaemia such as muscle spasms and tingling, as well as all other symptoms registered during the TPE cycle. We studied the number of patients experiencing one or more symptoms during a cycle, the total number of TPE sessions complicated by symptoms, and the duration of the symptoms. Subsequently, we investigated the association between kidney function and the occurrence of symptoms during or immediately after a TPE session. Lastly, we studied the occurrence of hyperkalaemia (plasma potassium concentration > 5.5 mmol/L) during the TPE cycle and the number of cases requiring medical intervention due to metabolic acidosis following TPE.

### 2.5 Statistical Analysis

Baseline characteristics and laboratory results are expressed as mean ± standard deviation (SD) for variables with a normal distribution and median ± interquartile range for variables with a non‐normal distribution. Categorical variables are presented as counts and percentages. Continuous data were analyzed using ANOVA or the Kruskal–Wallis test, whereas Pearson's chi‐squared tests or Fisher's exact tests were used to analyze categorical data.

Missing values in plasma electrolytes were imputed using the last measurement carried forward method. The magnitude of change in plasma electrolytes following a single TPE session was analyzed using a paired t‐test, and the difference among various eGFR groups was studied using one‐way ANOVA. Linear mixed‐effects models were used to analyze the association between kidney function and changes in plasma electrolyte concentrations and AG during a cycle of 5 TPE sessions. We corrected for the intensity of the treatment, number of TPE sessions, exchanged volumes, oral bicarbonate supplementation, and we incorporated an interaction term for eGFR and TPE sessions. For the mixed‐effects model analyses, eGFR was introduced as a continuous variable and each subject was introduced as a random effect. We used a generalized estimating equation to analyze the association between kidney function and the occurrence of symptoms during or after a TPE session. All analyses were performed using R version 4.3.2. The R packages used were Matrix, lme4, ggplot2, ggeffects, tidyverse, and tableone. All figures were created in R version 4.3.2 and GraphPad Prism 10.2.0.

## 3 Results

### 3.1 Study Population

Data from 121 patients were reviewed. In our analysis, we included 320 TPE sessions of 64 patients (Figure S1). The mean age was 50 years, 55% of the patients were male, and the average eGFR was 35 mL/min/1.7 3m^2^ (IQR 16–95). Baseline bicarbonate and chloride levels were 21 and 103 mmol/L, respectively (Table 1). Most patients (67%) had a renal indication to start TPE treatment. Eight patients were already receiving oral bicarbonate supplementation prior to the start of the TPE cycle. The mean volume replaced per session was 3.1 L (SD 0.4).

**TABLE 1:** Patient characteristics.

	eGFR groups (mL/min/1.73 m^2^)				
Characteristics^a^	Overall (*n* = 64)	≤ 19 (*n* = 21)	20–53 (*n* = 21)	≥ 54 (*n* = 22)	*p*
Sex—male (%)	35 (55)	12 (57)	10 (48)	13 (59)	0.82
Inpatient setting (%)	15 (23)	3 (14)	9 (43)	3 (14)	0.05
Age (years)	50 (16)	56 (15)	44 (14)	50 (19)	0.08
eGFR (mL/min/1.73 m^2^)	35 (16, 95)	13 (9, 16)	35 (29, 42)	104 (95, 122)	< 0.001
Number of TPE/day	0.70 (0.64, 0.70)	0.70 (0.70, 0.70)	0.70 (0.70, 0.70)	0.64 (0.63, 0.70)	0.005
Total number of TPE	7 (6, 7)	7 (7, 7)	7 (7, 7)	5 (5, 6)	< 0.001
Weight (kg)	81 (18)	84 (17)	77 (15)	83 (21)	0.37
Exchanged volume (L)	3.1 (0.4)	3.2 (0.3)	3.1 (0.5)	3.1 (0.3)	0.74
TPE indication (%)					
Nephrological	43 (67)	21 (100)	21 (100)	1 (4.5)	< 0.001
Neurological	20 (31)	0 (0)	0 (0)	20 (91)	
Dermatological	1 (2)	0 (0)	0 (0)	1 (4.5)	
Plasma [Na^+^] (mmol/L)	137 (4)	135 (5)	137 (3)	138 (3)	0.021
Plasma [Cl^−^] (mmol/L)	103 (6)	101 (8)	105 (4)	103 (3)	0.036
Plasma [HCO_3_^−^] (mmol/L)	21 (4)	19 (3)	20 (3)	24 (4)	< 0.001
Anion gap (mmol/L)	8 (3)	11 (3)	7 (3)	6 (2)	< 0.001
Oral bicarbonate supplementation (%)	8 (13)	5 (24)	2 (10)	1 (5)	0.15

Abbreviations: [Cl^−^], chloride concentration; eGFR, estimated glomerular filtrate rate; [HCO_3_^−^], bicarbonate concentration; [Na^+^], sodium concentration; *p*, *p* value for between group difference; TPE, therapeutic plasma exchange.

^a^ *N* (%) for categorical variables, mean (SD) for normally distributed continuous variables, median (IQR) for non‐normally distributed continuous variables.

Patients were stratified into tertiles according to their eGFR (≤ 19, 20–53, and ≥ 54 mL/min/1.73 m^2^). Plasma bicarbonate concentration differed significantly among the groups, with the lowest eGFR group having the lowest values (19 vs. 20 vs. 24 mmol/L; *p* < 0.001; Table 1). The plasma AG was significantly higher (*p* < 0.001), while the plasma sodium concentration was lower (*p* = 0.021) in those with eGFR ≤ 19 mL/min/1.73 m^2^. Baseline plasma chloride level was slightly higher in the middle eGFR group (*p* = 0.036). Patients in the lowest two eGFR groups had a more intensive TPE cycle than patients in the highest eGFR group.

### 3.2 Effect of a Single TPE Session

**FIGURE 1:**
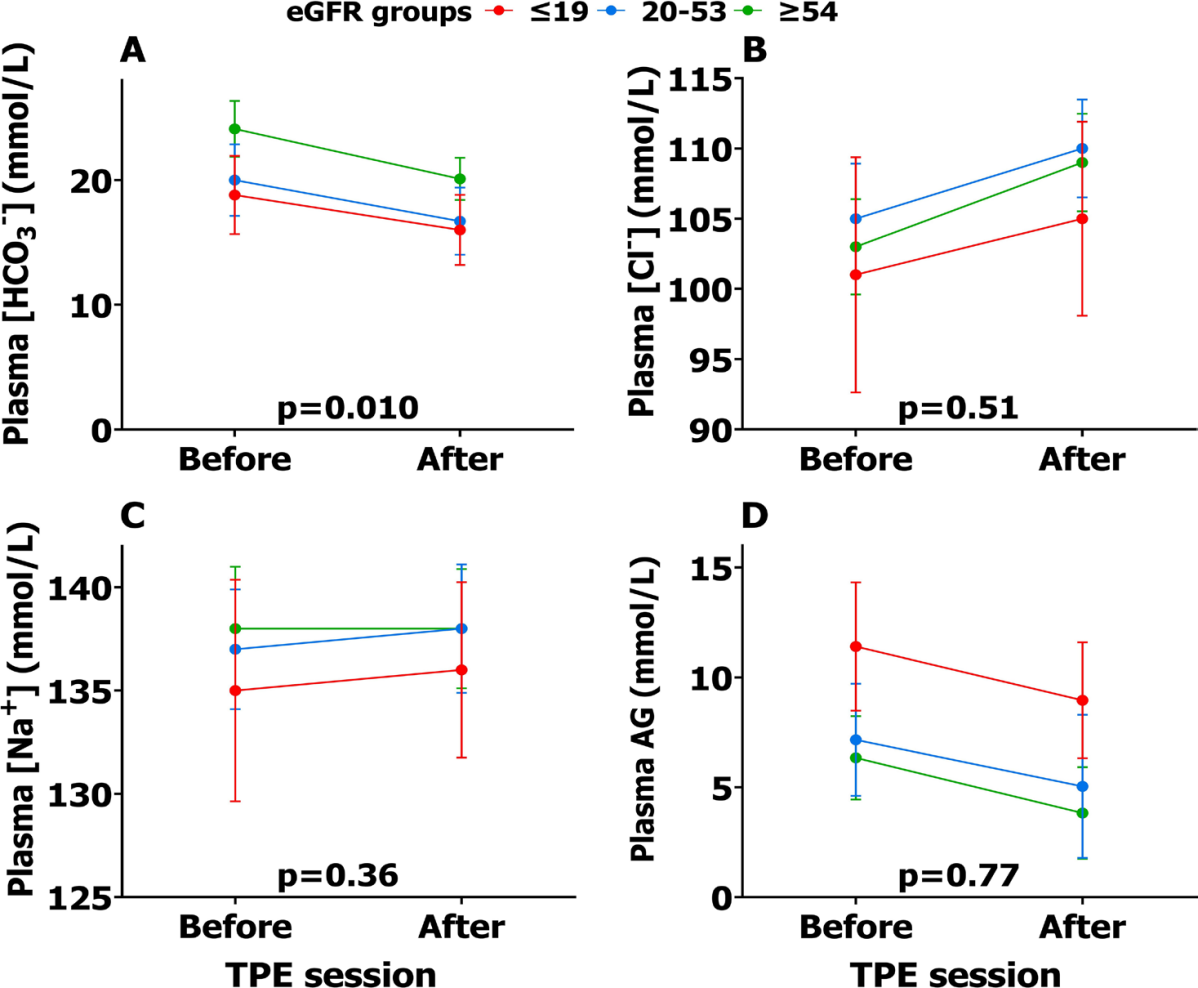
A single TPE session and electrolytes. Changes in plasma electrolytes (A‐C) and anion gap (D) following a single session of plasma exchange per eGFR group. Data are shown as mean ± SD. AG, anion gap; [Cl^−^], chloride concentration; eGFR, estimated glomerular filtration rate (mL/min/1.73 m^2^); [HCO_3_^−^], bicarbonate concentration; [Na^+^], sodium concentration; *p*, *p* value for between‐group differences using one‐way ANOVA; TPE, Therapeutic plasma exchange.

After the first TPE session, plasma bicarbonate concentration and AG decreased by 3.4 (*p* < 0.001) and 2.3 mmol/L (*p* < 0.001), respectively. Conversely, plasma chloride and sodium concentration increased by 5.0 (*p* < 0.001) and 0.8 mmol/L (*p* < 0.001). Similar changes were observed among all three eGFR groups, except for plasma bicarbonate concentrations, where patients in the highest eGFR group showed a greater decrease compared to those in the lower eGFR tertile groups (Figure 1, Table S1). The percentage change in plasma bicarbonate was similar across eGFR groups (*p* = 0.60).

### 3.3 Multiple Consecutive TPE Sessions

Plasma bicarbonate decreased and plasma chloride increased after multiple TPE sessions (Figure S2). After the fifth TPE, plasma bicarbonate concentration was 4.6 mmol/L lower (*p* < 0.001) than at baseline (Table S2). Plasma chloride concentration increased by 9.2 mmol/L (*p* < 0.001), and plasma sodium concentration increased by 2.5 mmol/L (*p* < 0.001) after five TPE sessions. The plasma AG was 3.7 mmol/L lower (*p* < 0.001) at the end of the fifth TPE compared to baseline.

**FIGURE 2:**
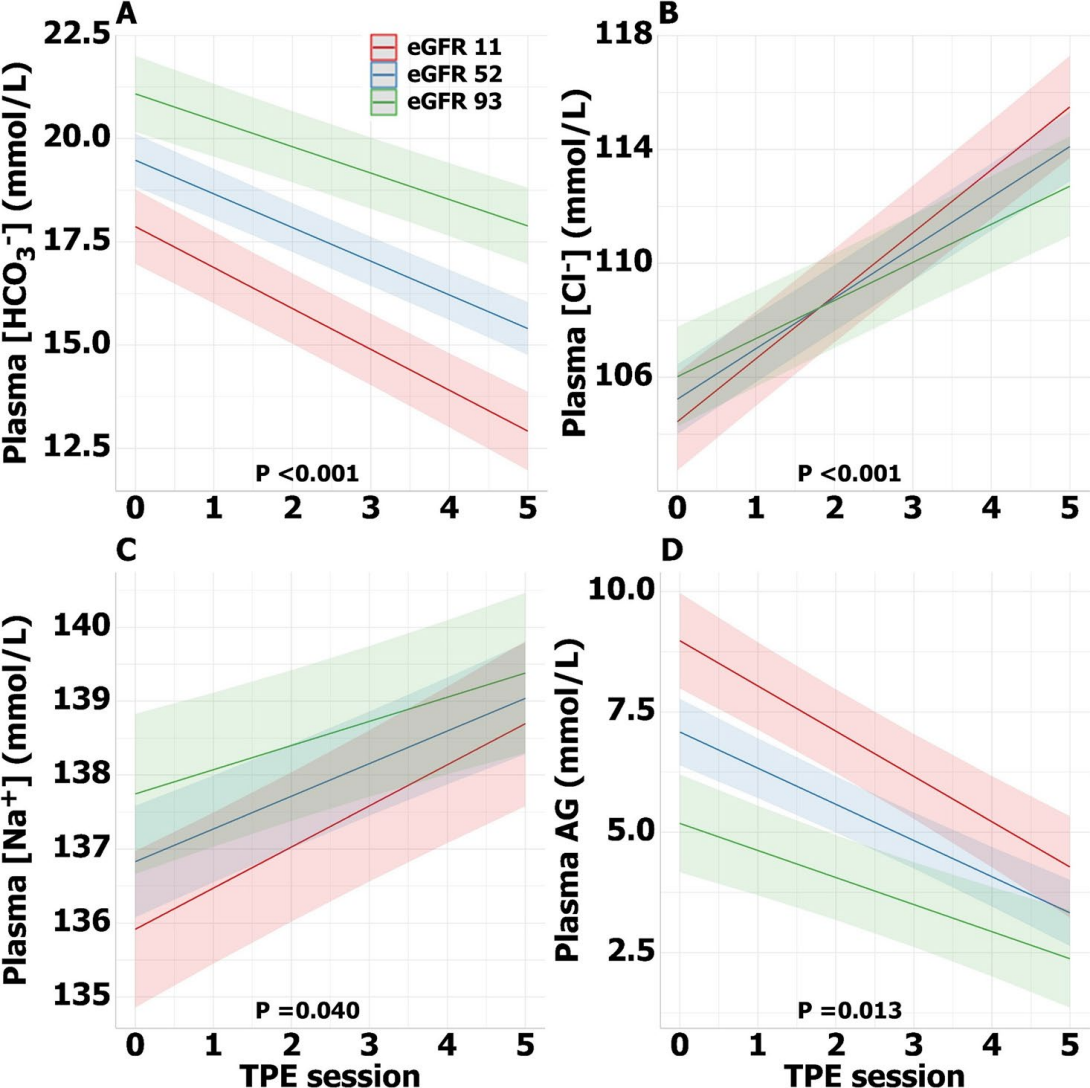
Effects of multiple consecutive TPE sessions. The association between plasma electrolyte concentration plotted against the two most important explanatory variables: Therapeutic plasma exchange session and eGFR. Interaction models for plasma bicarbonate (A), chloride (B), sodium concentration (C), and anion gap (D) using linear mixed‐effects models. AG, anion gap; [Cl^−^], chloride concentration; eGFR, estimated glomerular filtration rate (mL/min/1.73 m^2^); [HCO_3_^−^], bicarbonate concentration; [Na^+^], sodium concentration; *p*, *p* value corresponding to the difference between eGFR groups; TPE, therapeutic plasma exchange.

Our linear mixed‐effects model analysis revealed that the number of TPE sessions and the interaction between eGFR and TPE sessions were significantly associated with changes in plasma bicarbonate and chloride during the TPE cycle (Table 2). In individuals with lower eGFR, the decrease in plasma bicarbonate was more pronounced compared to those with normal kidney function (*p* < 0.001, Figure 2A). Similarly, plasma chloride levels increased substantially more in patients with lower eGFR than in those with normal kidney function (*p* < 0.001, Figure 2B). The decrease in plasma AG (*p* = 0.013) and the increase in plasma sodium concentration over time (*p* = 0.040) were also dependent on kidney function (Figure 2C,D, Table S3).

**TABLE 2:** Determinants of plasma chloride and bicarbonate changes.

	Chloride (*N* = 384)			Bicarbonate (*N* = 384)		
	Value	SE	*p*	Value	SE	*p*
Intercept	103.77	12.28	< 0.001	26.29	6.37	< 0.001
Session of TPE	2.32	0.14	< 0.001	−1.03	0.08	< 0.001
Intensity of TPE (session per day)	10.89	16.66	0.52	−12.78	8.55	0.14
eGFR (mL/min/1.73 m^2^)	0.02	0.02	0.20	0.04	0.01	< 0.001
Exchanged volume (L)	−0.00	0.00	0.035	−0.00	0.00	0.95
Dosage of oral bicarbonate supplementation (mg/day)	−0.00	0.00	0.36	0.00	0.00	0.47
eGFR × session of TPE	−0.01	0.00	< 0.001	0.00	0.00	< 0.001

*Note:* The association between various clinical parameters and changes in plasma chloride and bicarbonate concentration over time using linear mixed effect models.

Abbreviations: eGFR, estimated glomerular filtrate rate; *N*, number of observations included in the analysis for 64 patients; TPE, therapeutic plasma exchange.

### 3.4 Adverse Events

**FIGURE 3:**
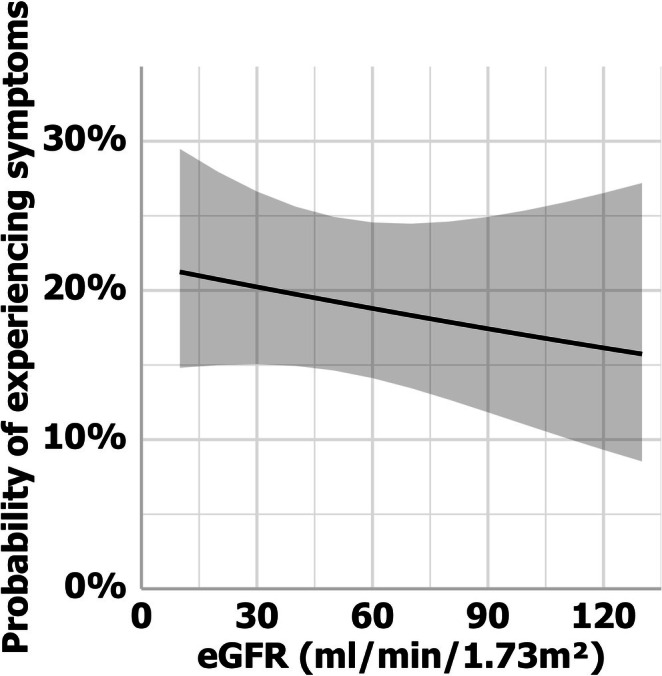
Association between kidney function and adverse events. The odds of a patient experiencing symptoms during or immediately after a TPE session based on their kidney function predicted using generalized estimating equations. The shaded area represents the 95% confidence interval. eGFR, estimated glomerular filtrate rate.

In general, patients tolerated a TPE cycle relatively well. Of all patients, 37 (56%) experienced one or more symptoms during this period. Of all 320 TPE sessions, 75 (23%) were complicated by ≥ 1 symptom(s). Among those who experienced symptoms, the median number of symptoms reported was 2 (IQR 1–3). The symptoms persisted for a median of 2 TPE sessions (IQR 1–3). eGFR was significantly associated with the odds of a patient experiencing symptoms during a TPE session (*p* = 0.004). With every 10‐unit decrease in eGFR, the odds of experiencing symptoms increased by a factor of 1.03 (Figure 3).

In addition to eight patients who used oral bicarbonate supplements at the start of the study, seven patients started with oral bicarbonate supplementation during the TPE cycle due to symptomatic metabolic acidosis. The average dose of oral bicarbonate supplements was 1.9 grams/day. All patients who initiated oral bicarbonate supplementation during the TPE cycle had an eGFR ≤ 32 mL/min/1.73 m^2^ and a plasma [HCO_3_^−^] below 15 mmol/L. The reason for starting oral bicarbonate supplementation varied among the patients (Table S4). In the original cohort of 121 patients, 12 patients received intravenous bicarbonate during the TPE cycle for treatment of metabolic acidosis.

In this cohort, only 1 TPE session was accompanied by a total‐to‐ionized calcium ratio > 2.5. Diarrhea was observed in five patients on one or more TPE days; three of these patients belonged to the lowest eGFR tertile, while the other two were in the middle tertile.

### 3.5 Hyperkalemia

Plasma potassium concentration during the TPE cycle was available in 23 patients with 115 measurements. The mean plasma potassium concentration was 4.1 ± 0.6 mmol/L. Hyperkalemia, defined as a plasma potassium concentration > 5.5 mmol/L, was observed in 1 patient in the lowest eGFR tertile group. No patient had a plasma potassium concentration > 6.0 mmol/L. Despite the occurrence of symptoms, mild hyperkalemia following TPE, and the need for medical interventions regarding plasma bicarbonate concentration, all patients completed their TPE cycle.

## 4 Discussion

In this prospective cohort study, we found that chronic kidney disease is an independent risk factor for the development of hyperchloremic metabolic acidosis after multiple TPEs with an albumin‐saline solution. This coincided with a higher rate of adverse events in this group.

We found that the effect of a single TPE on acid–base balance was similar across the eGFR spectrum, with the exception of plasma bicarbonate concentration. In all patients, plasma chloride concentration increased by 4–5 mmol/L. The absolute decrease in plasma bicarbonate concentration was greater in the highest eGFR tertile group. This can be attributed to the higher baseline plasma bicarbonate values, as the percentage change was similar across all eGFR groups. Our findings are in line with data from a previous study that investigated the acid–base changes occurring after TPE in 317 patients [1, 2]. This study focused on the effect of a single TPE session and did not assess the cumulative impact of multiple sessions on the observed acid–base changes. Additionally, the kidney function of these patients was not reported.

The impact of kidney function is best seen after multiple TPE sessions. To account for differences in TPE intensity across eGFR groups, TPE intensity was included as a covariate in the linear mixed‐effects models. Additionally, since patients with lower kidney function tended to undergo more TPE sessions compared to those with normal kidney function, potentially intensifying the acid–base disturbances, we limited our analysis to the first five sessions for all eGFR groups, regardless of the total number of sessions received. Whereas patients with normal kidney function were able to restore the acid–base disbalance before the next session, the metabolic acidosis became progressively worse in patients with impaired kidney function, with a mean plasma bicarbonate of 14 mmol/L in patients with an eGFR ≤ 19 mL/min/1.73 m^2^ after five TPE sessions.

Although the patients with impaired kidney function had a high AG metabolic acidosis at baseline, the newly acquired metabolic acidosis was of hyperchloremic origin as we observed a decrease in AG directly after each TPE session. Metabolic acidosis due to citrate accumulation during TPE was ruled out by a normal total calcium‐to‐ionized calcium ratio in > 99% of the TPE sessions. Metabolic acidosis secondary to hypotension was considered unlikely, as only one patient was hospitalized for sepsis, which was successfully treated with antibiotics and lactic acid concentrations were not increased. Other potential causes of metabolic acidosis, such as renal tubular acidosis, diarrhea, and diabetic ketoacidosis were unlikely as all changes occurred solely during the TPE session. The subsequent improvement in metabolic acidosis during the period between TPE further supports this conclusion.

The chloride content of the TPE replacement fluids is often unknown, and physicians may be unaware of the supraphysiologic chloride concentrations in these fluids, which are often referred to as “albumin solutions” instead of albumin‐saline solutions, which is more accurate. We now demonstrate for the first time that the high chloride content of these fluids has the potential to cause harm. Although the patients tolerated the TPE sessions relatively well, we observed that patients with impaired kidney function were more likely to experience symptoms during a TPE session compared to those with normal kidney function. These symptoms may not be recognized by the treating physician as the non‐specific symptoms can also be attributed to the underlying disease, such as renal transplant rejection.

The incidence of adverse symptoms in our study was relatively high compared to previous reports, which reported adverse event rates varying between 3% and 36% [2, 12, 13]. However, direct comparisons are challenging because prior studies included hemodynamic and biochemical changes, such as hypokalaemia, hypotension, and coagulation disorders, in their definitions of adverse events. In contrast, our study focused solely on patient‐reported symptoms. Additionally, most patients in earlier studies underwent less intensive TPE cycles and mostly for non‐renal indications. Notably, consistent with our results, Shemin et al. reported a higher incidence of nausea and vomiting among patients with focal sclerosing glomerulosclerosis (FSGS; 9%) compared to those receiving TPE for non‐renal indications (2%) [12]. However, similar to many other studies, Shemin et al. did not assess eGFR nor correlate the incidence of adverse events with eGFR. In a previous case report and in those cases that were excluded from our analysis due to intravenous bicarbonate infusion, we observed a quick improvement in symptoms after treatment, indicating that the metabolic acidosis itself might be causing these complaints [5]. However, we want to emphasize that not all cases of hyperchloremic metabolic acidosis during TPE require treatment with intravenous bicarbonate solutions. As acid–base disturbances are often mild when kidney function is preserved, treatment should only be considered in cases of severe metabolic acidosis and/or associated complaints.

Despite the severe metabolic acidosis in some subjects, we observed no severe or symptomatic hyperkalemia, possibly due to the fact that plasma exchange also lowers the total body potassium content [14, 15]. The long‐term effects of TPE‐induced hyperchloremic acidosis are unknown. Considering the similarities with the hyperchloremic metabolic acidosis that is seen after infusion of large volumes of saline in critically ill adults, one may speculate that the length of hospital stay, short and long‐term kidney function, and mortality of the patients receiving TPE could potentially be negatively affected by this metabolic disturbance [16, 17, 18, 19, 20].

A limitation of this study is that the laboratory values were available for the treating physician, who may have started oral bicarbonate supplements in patients with severe metabolic acidosis. Nevertheless, we still observed a steeper decline in plasma bicarbonate concentrations in patients with chronic kidney disease. Also, we had not standardized the registration of symptoms, which could introduce reporting bias and lead to an underestimation of the true frequency and variety of symptoms. The actual number of symptoms experienced could be higher than reported in this study.

In conclusion, we demonstrate for the first time that impaired kidney function is a risk factor for the development of hyperchloremic metabolic acidosis after multiple TPEs and is also associated with a higher incidence of self‐reported symptoms. Physicians should therefore be aware of the chloride concentration of the local albumin‐saline solutions and monitor the acid–base balance of patients with chronic kidney disease during an intensive TPE cycle.

## Author Contributions

R.H.O.E. and M.S.S. conceived the presented idea. S.S.A.S. and R.H.O.E. wrote the study protocol. S.S.A.S. informed and included the patients in this study. S.S.A.S. performed the statistical analysis and R.H.O.E. verified the analytical methods and assisted in interpreting the results. S.S.A.S. wrote the article under the supervision of R.H.O.E. and M.S.S.

## Conflicts of Interest

The authors declare no conflicts of interest.

## Supporting information


**Table S1:** Effect of one TPE on plasma electrolytes.
**Table S2:** Absolute changes in plasma electrolytes after five TPE sessions.
**Table S3:** Determinants of plasma sodium and anion gap changes.
**Table S4:** Indication for oral bicarbonate supplementation.
**Figure S1:** Flow diagram with included and excluded patients.
**Figure S2:** Changes in plasma electrolytes over time.

## Data Availability

The data that support the findings of this study are available on request from the corresponding author. The data are not publicly available due to privacy or ethical restrictions.
