# Supplementary material for: Chronic Kidney Disease Is a Risk Factor for the Development of Hyperchloremic Metabolic Acidosis After Repeated Therapeutic Plasma Exchanges
Source: J Clin Apher. 2025 Oct 7;40(5):e70059. doi: 10.1002/jca.70059 (PMC12504853; doi:10.1002/jca.70059)
Supplement: Supplementary file 1 — Table S1: Effect of one TPE on plasma electrolytes. Table S2: Absolute changes in plasma electrolytes after five TPE sessions. Table S3: Determinants of plasma sodium and anion gap changes. Table S4: Indication for oral bicarbonate supplementation. Figure S1: Flow diagram with included and excluded patients. Figure S2: Changes in plasma electrolytes over time. [file JCA-40-e70059-s001.pdf]

SUPPLEMENTARY DATA

Chronic kidney disease is a risk factor for the development of hyperchloremic metabolic acidosis after repeated therapeutic plasma exchanges

Authors: Sanédy SA Simon MD[1 2], Marit S van Sandwijk MD PhD[1 3], Rik HG Olde Engberink MD PhD[1 2]

[1]Amsterdam UMC location University of Amsterdam, Nephrology, Meibergdreef 9, Amsterdam, The Netherlands

[2]Amsterdam Cardiovascular Sciences, Diabetes and Hypertensive Diseases, Amsterdam UMC, Amsterdam, The Netherlands

[3]Dianet Dialysis Centre, Amsterdam, Netherlands.

TABLE OF CONTENT

Table S1. Effect of one TPE on plasma electrolytes 2
Table S2. Absolute changes in plasma electrolytes after 5 TPE sessions 2
Table S3. Determinants of plasma sodium and anion gap changes 2
Table S4. Indication for oral bicarbonate supplementation 3
Figure S1. Flow diagram with included and excluded patients 4
Figure S2. Changes in plasma electrolytes over time 5

Table S1. Effect of one TPE session on plasma electrolytes

	eGFR groups (ml/min/1.73m^2)				
	**Overall (n=64)**	**≤19 (n=21)**	**20-53 (n=21)**	**≥54 (n=22)**	**P**
Plasma [Na^+] (mmol/L)	0.83 (1.85)	0.90 (2.30)	1.19 (1.44)	0.38 (1.72)	0.36
Pasma [Cl^-] (mmol/L)	4.98 (2.36)	4.71 (2.74)	4.75 (2.17)	5.48 (2.14)	0.51
Plasma [HCO_3^-] (mmol/L)	-3.38 (1.31)	-2.83 (0.97)	-3.30 (1.39)	-4.02 (1.28)	0.010
Plasma AG (mmol/L)	-2.31 (2.35)	-2.48 (2.57)	-1.98 (2.91)	-2.45 (1.48)	0.77
Data are expressed at mean ±SD. P: p value for the between group difference. AG: anion gap; [HCO_3^-]: bicarbonate concentration; [Cl^-]: chloride concentration; eGFR: estimated glomerular filtrate rate; [Na^+]: sodium concentration					

Table S2. Absolute changes in plasma electrolytes after 5 TPE sessions

	eGFR groups (ml/min/1.73m^2)				
	**Overall (n=64)**	**≤19 (n=21)**	**20-53 (n=21)**	**≥54 (n=22)**	**P**
Δ Plasma [Na^+] (mmol/L)	2.48 (3.61)	3.24 (5.04)	2.48 (3.30)	1.71 (1.71)	0.40
Δ Pasma [Cl^-] (mmol/L)	9.21 (4.90)	11.90 (5.20)	8.71 (5.05)	7.00 (2.95)	0.003
Δ Plasma [HCO_3^-] (mmol/L)	-4.64 (2.18)	-4.69 (2.35)	-5.04 (2.06)	-4.19 (2.15)	0.45
Δ Plasma AG (mmol/L)	-3.70 (5.00)	-5.86 (3.19)	-2.81 (7.25)	-2.49 (2.90)	0.06
Data are expressed at mean ±SD. P: p value for the between group difference. AG: anion gap; [HCO_3^-]: bicarbonate concentration; [Cl^-]: chloride concentration; eGFR: estimated glomerular filtrate rate; [Na^+]: sodium concentration					

Table S3. Determinants of plasma sodium and anion gap changes

	**Sodium (N=384)**			**Anion gap (N=384)**		
	Value	SE	p	Value	SE	p
(Intercept)	139.56	7.54	<0.001	8.62	6.49	0.18
Session of TPE	0.59	0.09	<0.001	-0.99	0.12	<0.001
Intensity of TPE (session per day)	-0.37	10.18	0.97	-1.28	8.47	0.88
eGFR (ml/min/1.73m^2)	0.02	0.01	0.018	-0.05	0.01	<0.001
Exchanged volume (L)	-0.00	0.00	0.09	0.00	0.00	0.44
Dosage of oral bicarbonate supplementation (mg/day)	-0.00	0.00	0.29	0.00	0.00	0.98
eGFR*Session of TPE	-0.00	0.00	0.040	0.00	0.00	0.013
The association between various clinical parameters and changes in plasma sodium concentration and anion gap over time using linear mixed-effects models. eGFR: estimated glomerular filtrate rate; N: number of observations included in the analysis for 64 patients; TPE: therapeutic plasma exchange						

Table S4. Indication for oral bicarbonate supplementation

Patient	Reason for starting bicarbonate suppletion	Plasma [HCO_3^-] at the moment suppletion was started (mmol/L)
29	Headache	11.8
32	Muscle weakness	14.9
46	Extreme fatigue	13.2
53	Hyperkalaemia of 5.2 mmol/L	12.6
56	Metabolic acidosis during TPE cycle	11.2
62	Extreme fatigue	12.5
114	Hyperkalaemia of 5.6 mmol/L	14.5

Overview of the patients that started oral bicarbonate supplementation due to adverse events during TPE cycle. [HCO_3^-]: plasma bicarbonate concentration

Figure S1. Patient inclusion and exclusion

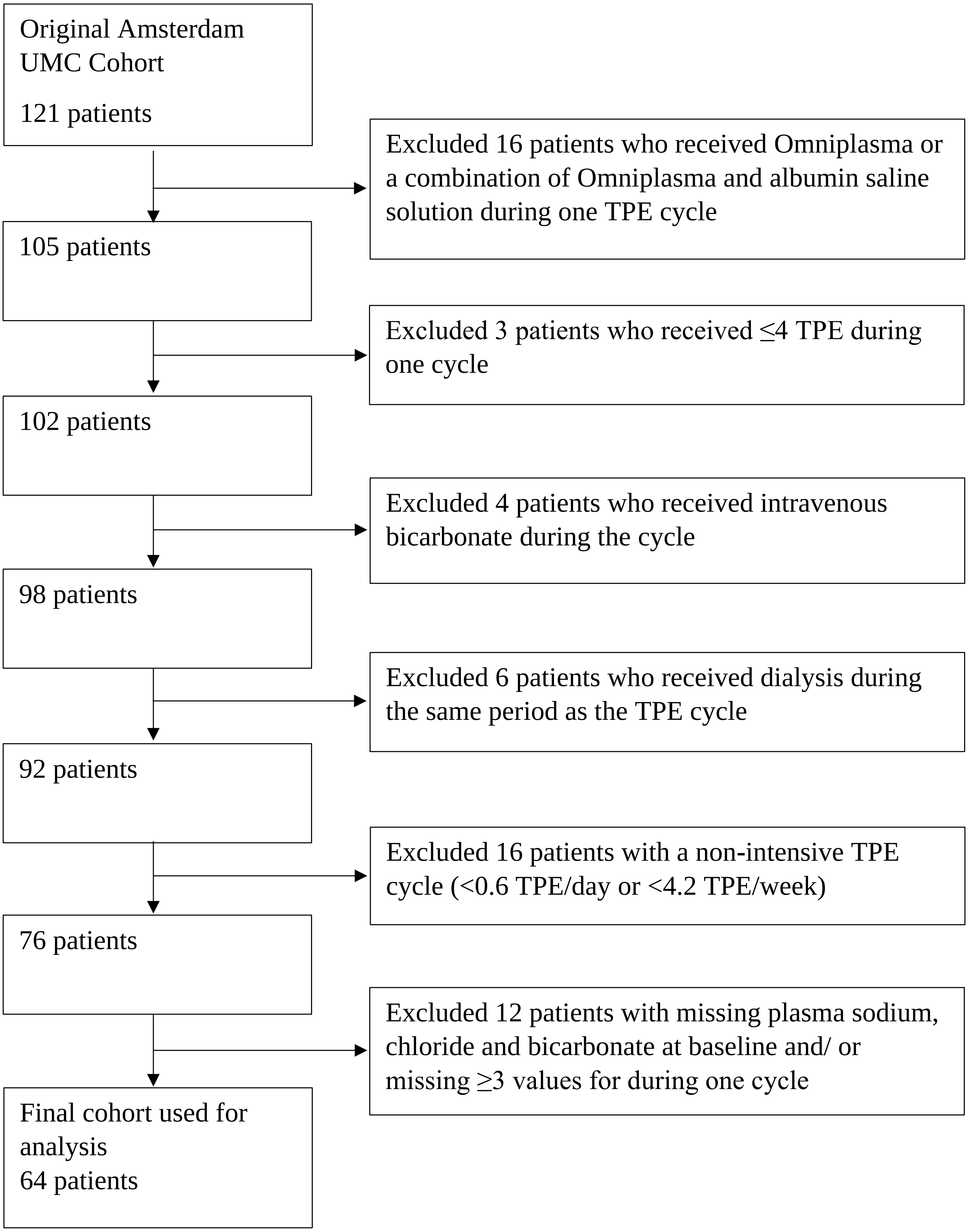

Flow diagram displaying the included and excluded patients and reason for exclusion.

Figure S2. Changes in plasma electrolytes over time.

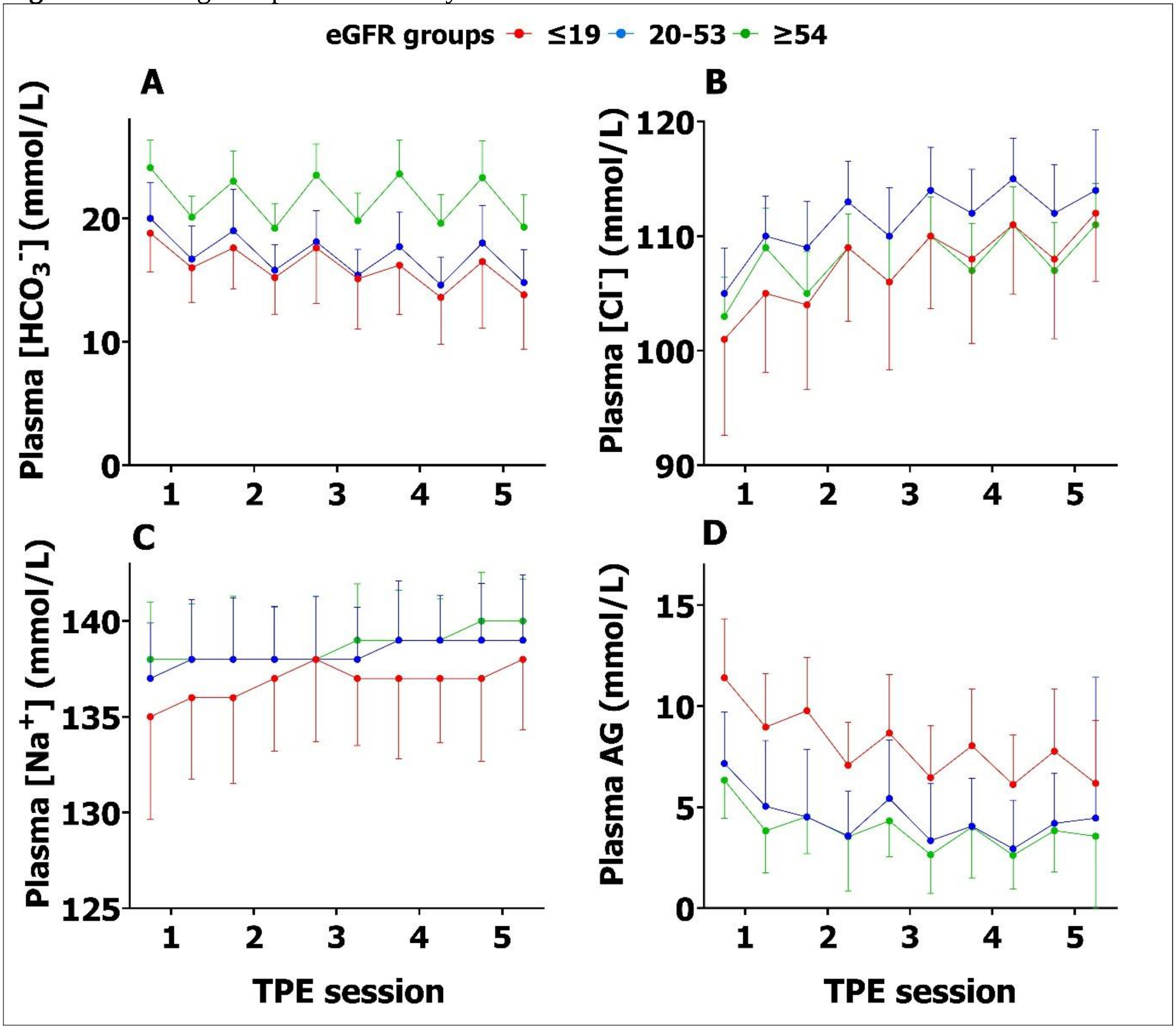

The unadjusted changes in plasma electrolytes concentration during a TPE cycle per eGFR group. Changes in mean plasma bicarbonate (A), chloride (B), sodium concentration (C) and anion gap (D). Data are presented as mean ±SD. AG: anion gap; [HCO_3^-]: bicarbonate concentration; [Cl^-]: chloride concentration; eGFR: estimated glomerular filtrate rate; [Na^+]: sodium concentration; TPE: therapeutic plasma exchange
